# Contemporary levels of cardiopulmonary resuscitation training in Denmark

**DOI:** 10.1016/j.resplu.2022.100268

**Published:** 2022-07-01

**Authors:** Anne Juul Grabmayr, Linn Andelius, Nanna Bo Christensen, Fredrik Folke, Kristian Bundgaard Ringgren, Christian Torp-Pedersen, Gunnar Gislason, Theo Walther Jensen, Astrid Rolin Kragh, Mads Christian Tofte Gregers, Julie Samsoee Kjoelbye, Carolina Malta Hansen

**Affiliations:** a. Emergency Medical Services Copenhagen, Telegrafvej 5, 2750 Ballerup, Denmark; b. Department of Clinical Medicine, University of Copenhagen, Blegdamsvej 3B, 2200 Copenhagen, Denmark; c. Department of Cardiology, Herlev Gentofte University Hospital, Gentofte Hospitalsvej 1, 2900 Hellerup, Denmark; d. Department of Cardiology, Aalborg University Hospital, Horovej 18-22, 9100 Aalborg, Denmark; e. Department of Cardiology, North Zealand Hospital, Dyrehavevej 29, 3400 Hillerød, Denmark; f. Department of Public Health, University of Copenhagen, Denmark; g. Department of Cardiology, Rigshospitalet, Blegdamsvej 9, 2100 Copenhagen, Denmark

**Keywords:** Out-of-hospital cardiac arrest, Cardiopulmonary resuscitation training, Automated external defibrillators, Volunteer responders, Resuscitation, OHCA, Out-of-hospital cardiac arrest, CPR, Cardiopulmonary resuscitation, AED, Automated external defibrillator

## Abstract

**Aim:**

Many efforts have been made to train the Danish population in cardiopulmonary resuscitation (CPR) and automated external defibrillator (AED) use. We assessed CPR and AED training levels among the broad Danish population and volunteer responders.

**Methods:**

In November 2018, an electronic cross-sectional survey was sent to (1) a representative sample of the general Danish population (by YouGov) and (2) all volunteer responders in the Capital Region of Denmark.

**Results:**

A total of 2,085 people from the general population and 7,768 volunteer responders (response rate 36%) completed the survey. Comparing the general Danish population with volunteer responders, 81.0% (95% CI 79.2–82.7%) vs. 99.2% (95% CI 99.0–99.4%) *p* < 0.001 reported CPR training, and 54.0% (95% CI 51.8; 56.2) vs. 89.5% (95% CI 88.9–90.2) *p* < 0.001 reported AED training, at some point in life.

In the general population, the unemployed and the self-employed had the lowest proportion of training with CPR training at 71.9% (95% CI 68.3–75.4%) and 65.4% (95% CI 53.8–75.8%) and AED training at 39.0% (95% CI 35.2–42.9%) and 34.6% (95% CI 24.2–46.2%), respectively.

Applicable to both populations, the workplace was the most frequent training provider. Among 18–29-year-olds in the general population, most reported training when acquiring a driver's license.

**Conclusions:**

A large majority of the Danish population and volunteer responders reported previous CPR/AED training. Mandatory training when acquiring a driver's license and training through the workplace seems to disseminate CPR/AED training effectively. However, new strategies reaching the unemployed and self-employed are warranted to ensure equal access.

## Introduction

Bystanders to out-of-hospital cardiac arrest who have not previously been trained in cardiopulmonary resuscitation (CPR) and automated external defibrillators (AED) use are less likely to provide CPR and defibrillation.1.., 2.., 3.. Broad initiatives to train the population in CPR and AED use are recommended by the American Heart Association (AHA), the European Resuscitation Council (ERC), and the International Liaison Committee on Resuscitation as a long-term strategy to increase bystander intervention and survival after out-of-hospital cardiac arrest (OHCA).4., 5., 6. Since 2013, the Danish Resuscitation Council has conducted annual events during 'Restart a Heart Day' with activities and free CPR courses nationwide,7., 8. but it is not clear how these have translated into training in CPR and AED use among the broad population. Moreover, training in AED use was only integrated into the ERC curriculum in 2010.

Even though CPR training at least once before graduating middle school (age 13–16) became mandatory by law in 2005, less than 30% of students had completed training 8 years after passing the legislation.^9^ Further, although CPR training has been required to acquire a driver's license since 2009, a large proportion of the population already had a driver's license by 2009, and 12% of the adult population does not hold a driver's license.10., 11., 12. A recent study reported that 44% of the Danish population attended a certified basic life support (BLS) course from 2009 to 2020, and about half of them when acquiring a driver's license.^11^ However, certified BLS courses are costly. It is essential to understand whether and how the broad population has access to CPR/AED training to prevent inequality in access to CPR/AED training.

In 2017, the Capital Region of Denmark implemented a volunteer responder program that activates registered volunteers to attend nearby OHCAs through a mobile phone app. It is not required to have completed CPR training before registration with the program, but strongly recommended. By November 2018, more than 20,000 people had registered as volunteer responders in the region, of whom approximately 25% registered as health care professionals.^13^

We hypothesized that the current mandated initiatives are not sufficient to ensure broad training of the current population in CPR and AED use. We aimed to investigate what proportion of the Danish population has ever received any CPR and/or AED training through a cross-sectional survey. To understand how the population has access to CPR and/or AED training, we also investigated the training provider. Lastly, we sought to compare findings from the general population with the population of volunteer responders.^13^

## Methods

### Study design

This cross-sectional questionnaire survey assessed two distinct populations, the general population in Denmark (general population survey) and the population of registered volunteer responders in the Capital Region of Denmark (volunteer responder survey). The minimum age in both populations was 18 years.

When signing up with the volunteer responder program, information regarding occupation is registered, and the responder can choose between four categories: health care professional, police/firefighter/ambulance personnel, student, or 'other'. This information was not reported among the general population.

No ethical approval was needed, as it is not mandated by law for this kind of survey in Denmark.

### Surveys

The cross-sectional questionnaire survey contained four questions; if and when the participants last received training in CPR or AED use; where they had received the training; and if they had ever participated in the resuscitation of a person. For example, respondents were asked: 'Have you ever received training or instructions in CPR?' or 'Have you ever received training or instructions in using a defibrillator?' One of the answer options was 'Yes, within the last year'. The survey did not include details about the quality of training, such as certification status, type (hands-on, online, etc.), or duration. The complete survey is displayed in Supplemental Material.

Participants could choose one or more of the following options for training providers: workplace, non-governmental organization (NGO), when acquiring a driver's license, leisure activity, military, primary school, secondary school, boarding school, high school, university, other, and do not remember. Volunteer responders had the additional opportunity to respond to this question as free text in contrast to the general population, who could only choose between the given options. We combined the educational options into three groups being primary (primary school), secondary (secondary school, high school, or boarding school), or higher education (university or college).

The respondents were divided into age groups, the youngest age group (18–29 years) being those who were likely to have been exposed to mandatory BLS training.

### The general population survey

In 2018, the population of Denmark was 5.7 million inhabitants with a median age of 41.8 years and 50.2% were female.^14^ The general population survey was an online market research survey of a representative sample of the Danish population. The survey was conducted by YouGov using active sampling among a nationally representative sample from the Danish YouGov Panel with more than 90,000 inhabitants in Denmark above 18 years of age. YouGov is an internet-based market research and data analytics firm. The methodology is well renowned for conducting representative internet-based surveys for research purposes^15^ and has previously been used in resuscitation science research.16., 17. The panel members are carefully recruited to represent the Danish adult population. They are selected to participate in surveys, so participants reflect the general population. Once they indicate that they are ready to answer, they receive the survey by email. Therefore, YouGov does not operate with response rates. Panel members receive gift cards for their participation in surveys (all respondents respond to all questions).^18^ Approximately 2,000 participants were needed to produce a margin of error of 2%, preventing a random sample error. A further increase in sample size was considered only to result in a diminishing improvement in the margin of error. Therefore, the sample size was set to 2,000 participants. As described by Statistics Denmark, distributions of age, sex, and geography have been weighted to represent the Danish population. The survey was conducted from November 2 to November 7, 2018.

### The volunteer responder survey

In 2018, the Capital Region of Denmark had a population of ∼1.8 million people and covered an area of 2,559 square kilometers.^14^ We conducted the volunteer responder survey among all 21,523 volunteer responders registered in the Capital Region of Denmark by November 8, 2018. We contacted the volunteer responders through a text message containing a link to the survey. Volunteer responders who did not answer the survey received a reminder after 24 hours.

### Statistical analysis

We compared the proportion of people who had reported completed training in CPR and AED use at some point in their lives and the source of training among the general population and volunteer responders. Since age did not follow a normal distribution, a Kruskal-Wallis test for non-parametric data was used to compare age between groups. Fisher's exact test was used for all categorical variables. The two populations were subdivided into age groups to examine if training providers depended on age. We used Cochrane Armitage Trend Test to analyze a trend in receiving CPR or AED training across age groups. Further, the general population was divided into types of occupations being unemployed, student/trainee, salaried professional, manual laborer, self-employed, and other.

Among volunteer responders, we compared those who responded to the survey with those who did not respond to examine the risk of non-response bias. Due to active sampling, this was not relevant for the general population survey. R statistics software was used for data analysis and figures.^19^

## Results

A total of 2,085 responded to the general population survey, and 8,020 out of 21,523 completed the volunteer responder survey (Table 1). Among volunteer responders, 252 were excluded due to missing answers resulting in 7,768 included responders among volunteer responders (response rate of 36%).

**Table 1.** Baseline characteristics of participants in the general population and the volunteer responder population.

Variable	General population (*n* = 2,085)	Volunteer responders (*n* = 7,768)
Age, median (Q1, Q3)	49 (33, 63)	39 (29, 50)
Sex (female), *n* (%)	1,063 (51)	3,962 (51)
Health care professional, *n* (%)	Unknown	2,563 (33)
Police, firefighter, and ambulance personnel, *n* (%)	Unknown	621 (8)
Student, *n* (%)	250 (12)	1,088 (14)

### CPR and AED training

**Fig. 1.**
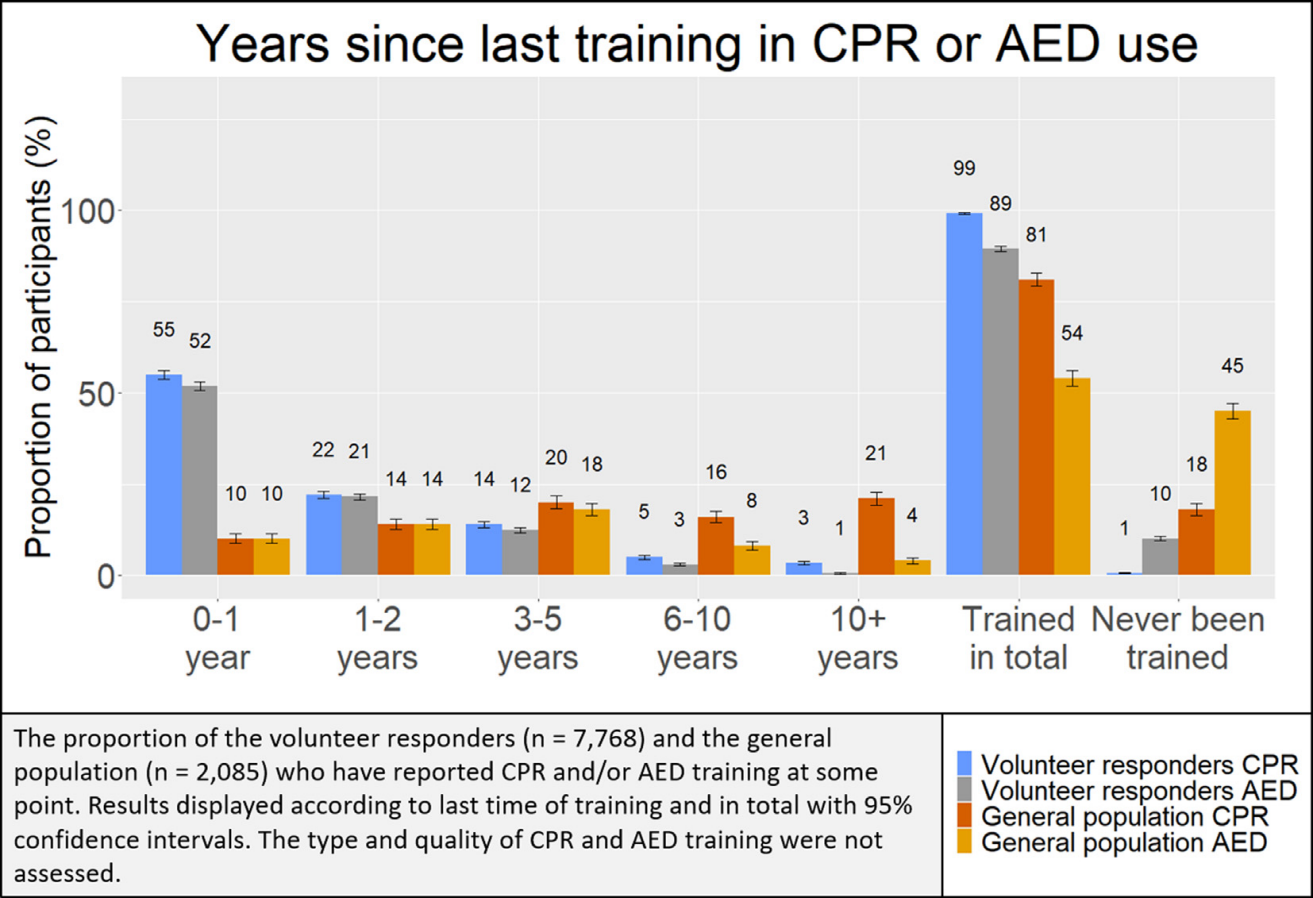
Years since participants last received training in CPR and AED use according volunteer responder and general population.

The majority of both populations reported previous training in CPR and AED use though volunteer respondents reported significantly higher percentages than the general population in training in CPR (99% vs. 89%, *p*-value <0.001) and AED use (81% vs. 54%, *p*-value <0.001). Fig. 1 shows the distribution of time since last training in the two populations. Compared with the general population, a significantly higher proportion of volunteer responders reported training within recent years. Most volunteer responders reported training within the last two years, whereas most reported training >10 years prior to receiving the survey among the general population.

### CPR and AED training across age groups and types of occupation

**Fig. 2.**
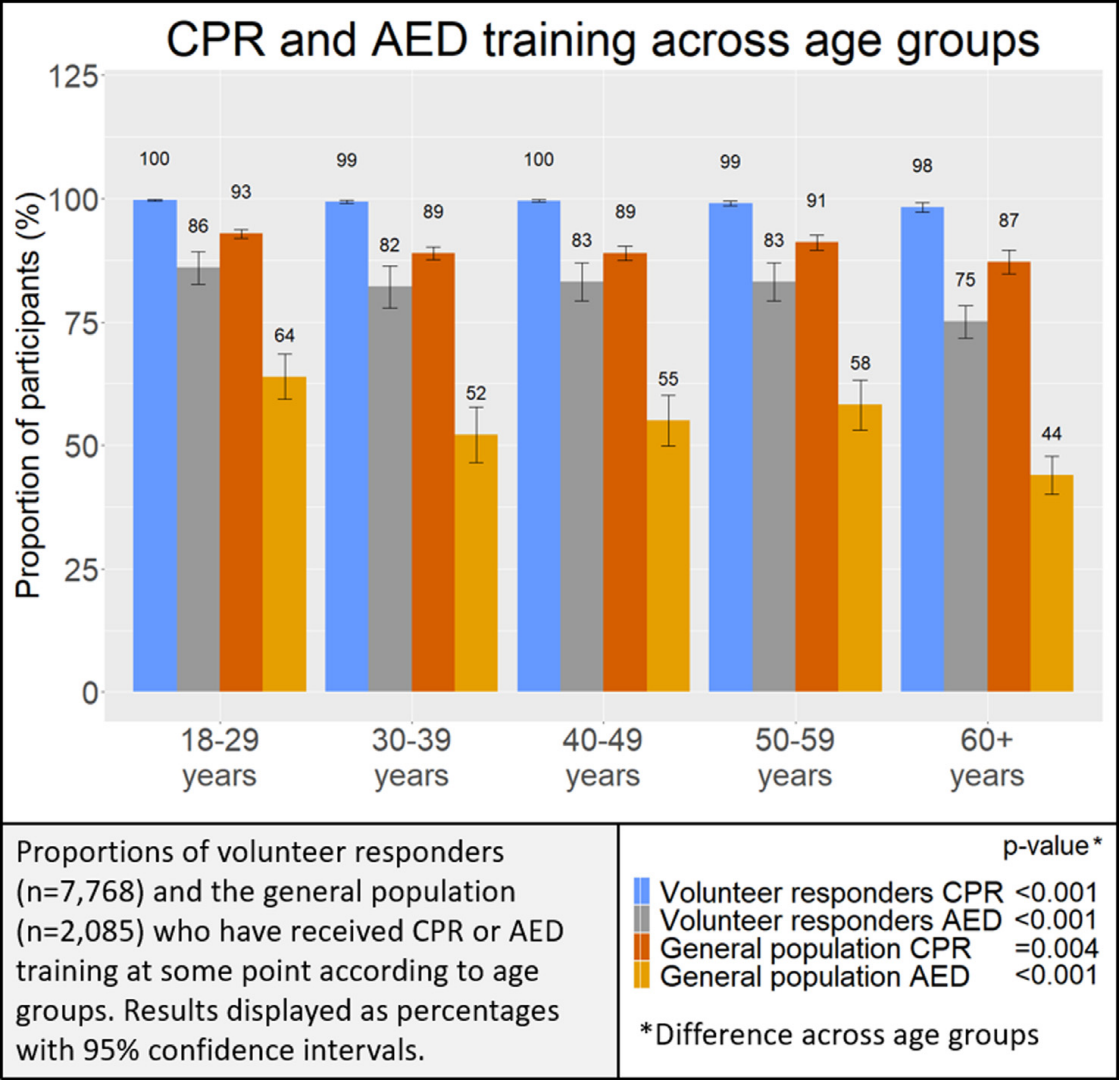
CPR and AED training according to age groups among volunteer responders and the general population.

As shown in Fig. 2, volunteer responders reported significantly more training than the general population across all age groups. We found a statistically significant difference between age groups when analyzing CPR training and training in AED use among the general population. The difference across age groups was also significant among volunteer responders regarding CPR training and training in AED use, indicating that the younger age groups had received more training than the older age groups.

When divided into types of occupation, as shown in Table 2, manual laborers reported a higher level of training than all other groups, especially the unemployed, self-employed, and others have low levels of CPR and AED training.

**Table 2.** Training and resuscitation in the general population divided into occupation.

Item	Received CPR training *n* (%)	*p*-value	Received AED training *n* (%)	*p*-value
Manul laborers (*n* = 323)	297 (92)	Ref	216 (67)	Ref
Salaried proffessionals (*n* = 742)	631 (85)	**0.02**	445 (60)	**0.03**
Self-employed (*n* = 78)	51 (66)	**<0.001**	27 (35)	**<0.001**
Unemployed (*n* = 638)	459 (72)	**<0.001**	249 (39)	**<0.001**
Student/Trainee (*n* = 242)	208 (86)	**0.03**	145 (60)	0.09
Other (*n* = 62)	44 (71)	**<0.001**	30 (48)	**0.009**

CPR training in the general population divided into occupations. The groups have been compared using the manual laborers as the reference group.

### Training provider

**Fig. 3.**
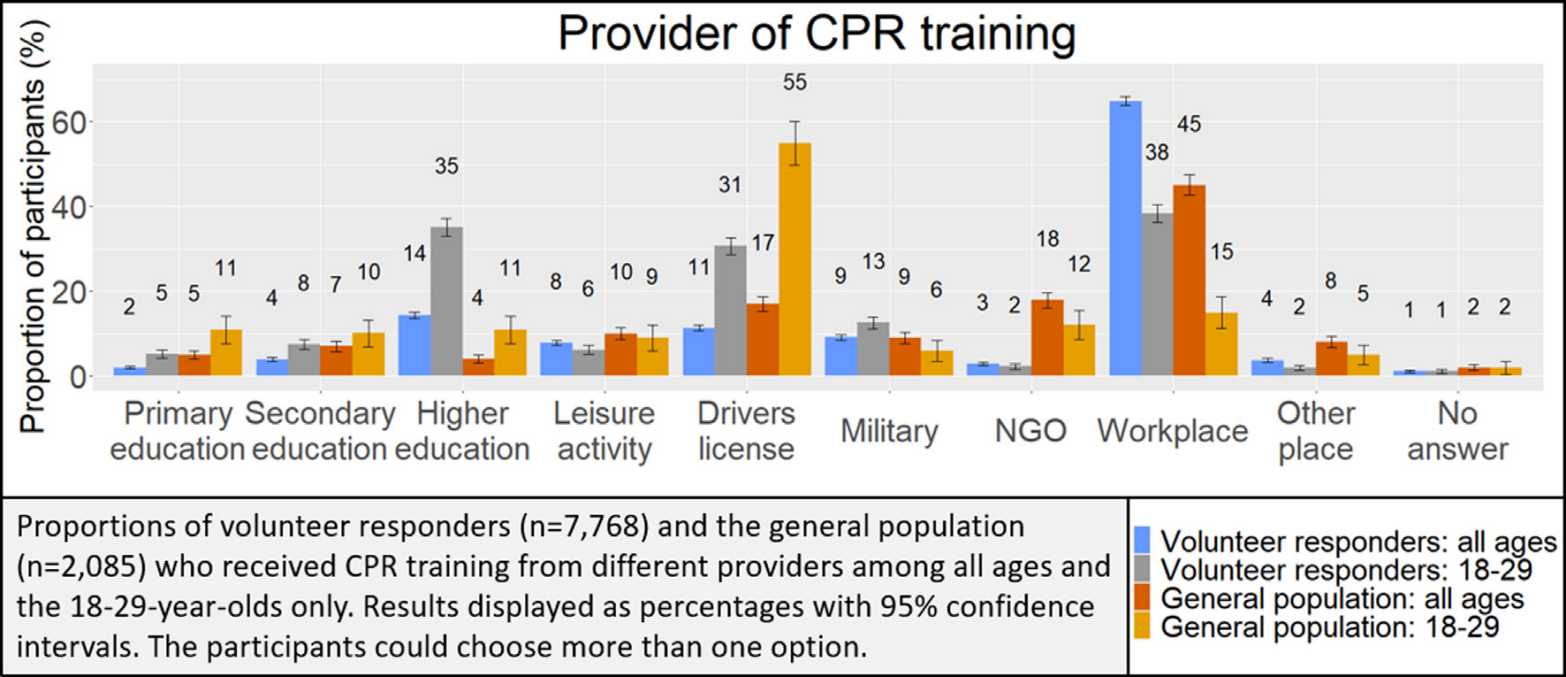
Providers of CPR training among volunteer responders and the general population for all age groups and among the 18–29-year olds.

Fig. 3 shows an overview of all providers of CPR and AED training. The main provider in both populations was the workplace. Among volunteer responders, the most common providers of CPR training following the workplace (65%) were higher education (14%), followed by when acquiring a driver's license (11%) and a military organization (9%). Among the general population, the most common venues for training following the workplace (45%) were NGOs (18%), when acquiring a driver's license (17%), and leisure activity (10%). When examining only the youngest age group, 18–29 years, the most common training provider was the workplace (38%), followed by higher education (35%) and when acquiring a driver's license (31%) for the volunteer responder population and when acquiring a driver's license (55%) for the general population.

### Non-respondents to the survey

As explained in the methods section, there were no non-respondents among the general population. For volunteer responders, baseline characteristics of non-respondents compared with respondents in the volunteer responder survey are shown in Supplemental Material Table a (there was no information for 11% of non-respondents). Non-respondents were significantly younger, less likely to be health care professionals, police, firefighter, or ambulance personnel, and more likely to be students. 99.2% of respondents and 98.6% of non-respondents had undergone CPR training at some point in time (*p*-value <0.001), and 77% vs. 65% had undergone training within two years before signing up for the program (*p*-value <0.001), respectively.

## Discussion

This cross-sectional study of contemporary CPR and AED training in a representative sample of the Danish population and the registered volunteer responder population in the Capital Region of Denmark had several main findings. Four out of five people in the Danish population and largely all (99%) registered volunteer responders reported previous CPR training. More than half of the general population and 90% of the volunteer responder population reported previous AED training. Most volunteer responders reported training within two years before the survey was conducted, whereas most people in the general population had received training more than 10 years before receiving the survey. Finally, the most common training provider among both populations was the workplace, followed by NGOs and when acquiring a driver's license among the general population, and higher education among volunteer responders. Among the general population in the age group 18–29 years, the most common training provider was when acquiring a driver's license. These findings suggest that CPR and AED training have successfully been implemented in Denmark, largely due to CPR/AED training provided by the workplace and not by mandated initiatives.

Our findings indicate that implementing mandatory CPR training when acquiring a driver's license is a successful way to ensure widespread CPR/AED training.^11^ However, reaching a large proportion of the population will take decades, underscoring the importance of providing training through other institutions. Further, not all people acquire a driver's license.^12^ We found that the workplace seems to be the most instrumental CPR/AED training provider among the adult Danish population, which is encouraging but inevitably excludes those who are not part of the workforce^20^ and the self-employed. This is supported by the lower proportion of CPR/AED training among self-employed and unemployed.^11^ These observations are important as neighborhoods with lower socioeconomic status have been associated with a lower likelihood of prior CPR training and bystander intervention.21., 22., 23., 24. It is thus paramount to ensure CPR/AED training across all working groups and the unemployed to prevent further socioeconomic disparity in care for out-of-hospital cardiac arrest patients. Training programs targeting students, retirees, the self-employed, and the unemployed could help counteract this.

Despite CPR courses being mandatory by middle school graduation in Denmark since 2005, respondents did not report high proportions of training in Middle School, even among the youngest age group (18–29 years). Thus, mandatory CPR training in schools has not yet had the intended effect of broadly reaching the population. This could be due to challenges with the implementation of CPR/AED training in Danish schools, as previously reported.9., 25., 26. Recall bias could also contribute to our findings.23., 27. Since this study was not designed to investigate the effect of CPR/AED training in middle schools, further research is needed to reevaluate the current status of CPR/AED training in Danish middle schools.

Many efforts are being made to ensure widespread CPR training across populations in many countries, but few countries have reported similarly high proportions of CPR training across their general population.20., 22., 27., 28. Our findings of widespread CPR training among the population are also supported by an overall increase in bystander CPR from 20% to 80% and bystander defibrillation 1.4–8.7% from 2001 to 2020.^29^ The highest reported proportion of CPR trained population is from Norway, where 90% had received training.^27^ In Norway, first aid has been part of the primary school curriculum since 1961, but only 64% of teachers include CPR in classes.^30^ Further, CPR training has been mandatory when acquiring a driver's license in Norway since 2003.^27^ In Australia, where there are no mandatory CPR courses, 56% of the population has been trained in CPR at some point in their lives.^28^ In the US, it has been reported that 65% of the population had received training at some point, but with low annual rates of training and great differences across the country.21., 22. In states with mandatory CPR training before high school graduation, 17% had been trained within the last two years vs. 14% in states without mandatory CPR training,21., 31. which supports mandatory training programs may have an impact on ensuring widespread CPR training. However, this has not been tested in a randomized trial.

Current resuscitation guidelines from the ERC and the AHA recommend frequent retraining every one-two years.4., 5. Importantly, a small part of the respondents among the general population reported training within the last two years in our study. This highlights the importance of providing regular access to CPR/AED training. Interestingly, most volunteer responders had received training within two years, indicating those who register with the volunteer responder program tend to have more recent training, which was not unexpected since 25% of all volunteer responders are healthcare professionals. Training through the workplace seems to be the most suitable training provider for frequent training. A recent study found that 3.6% of the Danish population had completed a certified BLS course annually, and 44% completed a certified BLS course from 2009 to 2019.^11^ In our study, 10% of the general population reported receiving training or instructions in CPR within the last year prior to the survey and 60% from 2008-2018. This suggests that people receive CPR/AED training from other sources other than the certified BLS courses and supports the high levels of CPR training in the Danish population identified in the present study. As a long-term strategy, aiming for training the population in middle school, high school, through driver's license acquisition, and then the workplace seems to be sustainable to ensure repetitive and frequent training. But further efforts are needed to reach the unemployed, self-employed and other groups that do not fall into the above categories.

## Limitations

The questionnaire was developed to screen the population for CPR/AED training and thus include all sources of instructions in CPR and AED use. This means that the survey concerns not only people who have undertaken a certified resuscitation course but also people who may have received instruction from a friend or a colleague, through video/e-learning material, or other sources. Therefore, this study was not designed to investigate the quality of resuscitation training or retention skills among the participants. The questionnaire was not pilot-tested or validated through cognitive interviewing.^32^ However, the instrument survey was assessed and modified by YouGov, a company with extensive expertise in this field. Further, item validation is not required since the survey did not include complex scales or measuring constructs.^33^

There is a risk of non-response bias since the volunteer responder survey had a fairly low response rate (36%), and there were differences in demographics between respondents and non-respondents. However, the risk of bias in terms of proportions trained seems unlikely since 99% of non-respondents have undergone training as reported per registration with the program and recently published.^13^ The general population survey does not have the risk of response bias since the participants only knows the subject of the survey once they agreed to participate.

## Conclusion

This cross-sectional study found that training in CPR and AED use is widespread among the general Danish population and volunteer responders. CPR and AED training through the workplace and when acquiring a driver's license seem to reach the population effectively. Still, new strategies are needed to ensure equal access to CPR training across all occupational groups.

## Conflicts of interest

The authors had no conflicts of interest to declare.

## Source of funding

Anne Juul Grabmayr has received a grant from TrygFonden. Fredrik Folke has received a grant from the Novo Nordisk Foundation (grant number: NNF19OC0055142).
