# Supplementary material for: Contemporary levels of cardiopulmonary resuscitation training in Denmark
Source: Resusc Plus. 2022 Jul 1;11:100268. doi: 10.1016/j.resplu.2022.100268 (PMC9256815; doi:10.1016/j.resplu.2022.100268)
Supplement: Supplementary data 1. [file mmc1.docx]

**Supplemental Material**

*Survey translated to English*

Q1 Have you ever received training or instructions in cardiopulmonary resuscitation (chest compressions and rescue breaths)?

1. Yes, within the last year
2. Yes, within the last 1-2 years
3. Yes, within the last 3-5 years
4. Yes, within the last 6-10 years
5. Yes, more than 10 years ago
6. No
7. Do not remember

Q2 Have you ever received training or instructions in using a defibrillator?

1. Yes, within the last year
2. Yes, within the last 1-2 years
3. Yes, within the last 3-5 years
4. Yes, within the last 6-10 years
5. Yes, more than 10 years ago
6. No
7. Do not remember

Q3 Where have you received training or instructions in resuscitation (chest compressions/rescue breaths/using a defibrillator)?

1. Primary school
2. Secondary school
3. Boarding school
4. Danish folk high school
5. When acquiring a drivers license
6. Military
7. University
8. Workplace
9. Leisure activities (scouts/swimming and similiar)
10. Non-governmental organizations
11. (11): Other (free text)

Q4 Have you ever participated in resuscitating a person?

1. Yes
2. No
3. Other

*Survey in Danish*

Q1 Har du nogensinde fået undervisning/instruktion i hjertelungeredning (hjertemassage og kunstigt åndedræt)?

1. Ja, inden for det seneste år
2. Ja, inden for de seneste 1-2 år
3. Ja, inden for de seneste 3-5 år
4. Ja, inden for de seneste 6-10 år
5. Ja, for mere end 10 år siden
6. Nej
7. Husker ikke.

Q2 Har du nogensinde fået undervisning/instruktion i brug af en hjertestarter?

1. Ja, inden for det seneste år
2. Ja, inden for de seneste 1-2 år
3. Ja, inden for de seneste 3-5 år
4. Ja, inden for de seneste 6-10 år
5. Ja, for mere end 10 år siden
6. Nej
7. Husker ikke.

Q3 Hvor har du fået undervisning/instruktion i hjertelungeredning (hjertemassage/kunstigt åndedræt/brug af hjertestarter)?

1. Folkeskole/grundskole
2. Gymnasium/HF/Teknisk skole/eux
3. Efterskole
4. Højskole
5. I forbindelse med erhvervelse af kørekort
6. Militæret
7. Universitet
8. Arbejdsplads
9. Fritidsaktivitet (spejder/svømning og lignende)
10. falck, røde kors eller lign.
11. andet

Q5 Har du nogensinde deltaget i genoplivning af en person?

1. Ja
2. Nej
3. Andet

**Table a** Baseline characteristics of non-responders and responders among the volunteer responder population.

| **Baseline characteristics of non-responding volunteer responders** | | | |
| --- | --- | --- | --- |
|  | Non-respondents  n = 12358  missing = 1396 | Respondents  n = 7768  missing = 0 | p-value |
| Age in years, median (Q1, Q3) | 35 (27, 47) | 39 (29, 50) | <0.001 |
| Sex (female), % (n) | 48.7 (6013) | 51.2 (3981) | <0.001 |
| Health care professional, % (n) | 23.7 (2924) | 32.8 (2551) | <0.001 |
| Police, firefighter, or ambulance personnel % (n) | 5 (500) | 8 (649) | <0.001 |
| Student, % (n) | 20 (2669) | 14 (1148) | <0.001 |
| Last trained in CPR |  |  |  |
| 0-1 year, % (n) | 38.2 (4724) | 53.3 (4141) | <0.001 |
| 1-2 years, % (n) | 26.6 (3291) | 23.8 (1847) | <0.001 |
| 2-5 years, % (n) | 30.8 (3808) | 19.1 (1484) | <0.001 |
| > 5 years, % (n) | 3.0 (367) | 3.0 (230) | 1.0 |
| Trained in CPR in total, % (n) | 98.6 (12190) | 99.2 (7702) | <0.001 |
| Never been trained in CPR, % (n) | 1.4 (168) | 0.8 (65) | 0.001 |

When registering as the volunteer responder, volunteer responders report age, sex, profession, if they have received training in CPR, and if yes, when they last received training. This data has been used to compare respondents and non-respondents among the volunteer responders.
